# Computational Psychiatry and Computational Neurology: Seeking for Mechanistic Modeling in Cognitive Impairment and Dementia

**DOI:** 10.3389/fncom.2022.865805

**Published:** 2022-05-11

**Authors:** Ludmila Kucikova, Samuel Danso, Lina Jia, Li Su

**Affiliations:** ^1^Department of Neuroscience, Sheffield Institute for Translational Neuroscience, Insigneo Institute for in silico Medicine, University of Sheffield, Sheffield, United Kingdom; ^2^Edinburgh Dementia Prevention and Centre for Clinical Brain Sciences, Edinburgh Medical School, University of Edinburgh, Edinburgh, United Kingdom; ^3^Beijing Anding Hospital, Capital Medical University, Beijing, China; ^4^Department of Psychiatry, School of Clinical Medicine, University of Cambridge, Cambridge, United Kingdom

**Keywords:** dementia, computational psychiatry, computational neurology, computational neuroscience, computational modeling, machine learning, artificial intelligence

## Introduction

The prevalence of dementia is increasing globally and carries a growing personal and societal burden (Guerchet et al., 2013). Multimodal and longitudinal neuroimaging provides biomarkers about disease progression and informs early detection of dementia (Ten Kate et al., 2018). However, current empirical data is still insufficient to infer the underlying mechanisms of the disorder necessary for developing targeted therapeutics. Equally important to the lack of empirical data, there is an absence of sufficient theoretical tools to investigate the relationships among the genetic risks, neuropathophysiology, clinical symptoms and environmental factors in neurodegenerative diseases.

We argue that the recent advances in computational psychiatry and computational neurology offer a promising translational neuroscience framework for integrating multiple levels of abstractions and investigating neurobiological and pathological mechanisms of dementia. In addition, they can derive mechanistic models that predict disease trajectory and treatment effects. Here, we extend historical discussions on this topic (Adams et al., 2016; Paulus et al., 2016; Hitchcock et al., 2022) by discussing the potential of integrative computational modeling for dementia research. We will discuss the potential translational benefits and how it might account for some of the current limitations in dementia research.

## Computational Psychiatry and Computational Neurology

With the rise of computational and data sciences applications in biological, medical, biomedical, and psychological disciplines since the early 2010s, computational psychiatry and computational neurology have demonstrated the potential to help account for some of the limitations of traditional techniques (Montague et al., 2012). Computational psychiatry and neurology are interwind and overlap in disorders like dementia, so in the context of this paper, we do not distinguish them.

There are several different dichotomies of computational psychiatry and neurology models (e.g., descriptive vs. predictive, discriminative vs. generative, exploratory vs. confirmatory models). We recognize that all these dichotomies have their value in describing specific classes of computational models. However, in this paper, we would like to make a distinction between two types of scientific research approaches by their different objectives: data-driven vs. theory-driven approaches. We argue that the former primarily aims to explain patterns in novel data or information “about” the diseased brain, while the latter primarily aims to develop, validate, or falsify theories which describe information of the brain.

### Data-Driven Computational Approaches

The primary objective of data-driven approaches is to “label” experimental data based on multivariate patterns or statistical regularities in the data (e.g., by using machine learning). An advantage of data-driven approaches is that they require minimal prior assumptions about the data (Magoulas and Pentza, 1999). However, this very characteristic also makes the interpretation of data-driven models challenging as discussed by Goecks and colleagues (Goecks et al., 2020).

The current applications of machine learning in dementia research focus on disease detection and prediction. For example, by using neuroimaging, biological, and clinical data based on recurrent neural networks, support vector machines, decision trees, Naïve Bayes classifiers, clustering, or other methods (Cui and Liu, 2019; Kuan et al., 2021; Skolariki et al., 2021). Moreover, the efforts were made to develop a personalized dementia risk model that could predict the onset of dementia years before patients develop symptoms by using ensemble learning from demographic and medical history data (Danso et al., 2021). Data-driven applications were also used to discriminate different types of dementia (Dauwan et al., 2016; Bougea et al., 2021) or to decrease the number of measures necessary for diagnosis (Weakley et al., 2015). Other computer-aided diagnosis systems that automatically detect neurological abnormalities have been developed for the identification of dementia from neuroimaging data (Siuly and Zhang, 2016). However, translating these efforts into clinical practice is still problematic and need more of easily used real-time methods that can be incorporated into everyday clinical practice.

Although, some examples that directly aim to model disease mechanisms, neuropathology, or subtypes exist (Young et al., 2014; Oxtoby et al., 2018; Su et al., 2018, 2021), majority of mainstream data-driven approaches do not explicitly intend to capture the neurobiological and neuropathological mechanisms underlying dementia. While applicable for disease prediction and diagnosis, data-driven approaches alone are still limited to inform novel treatments and capture the underlying complexities of dynamic nature of dementia (i.e., interactions of multiple disease factors on different levels that can evolve in complex ways over time).

### Theory-Driven Computational Approaches

**Figure 1:**
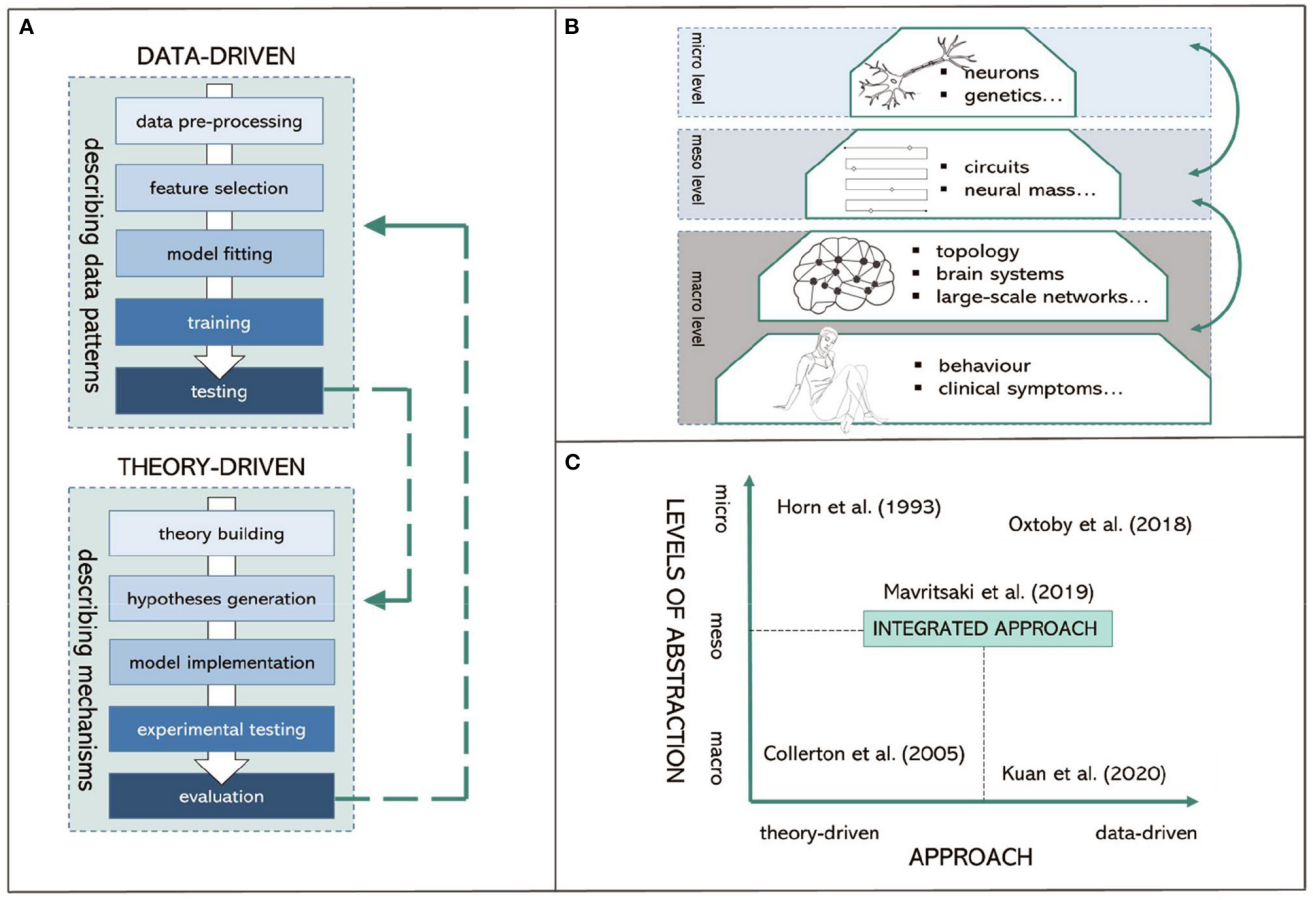
Visual summary of proposed integrated computational modeling. **(A)** Data-driven and theory-driven pipelines. **(B)** Different levels of abstraction. **(C)** Proposed integrated computational modeling with examples.

Theory-driven approaches for computational psychiatry and neurology are used to describe the mechanisms of altered pathology or information processing related to the “cause” of psychiatric or neurological conditions. They are used as tools for characterizing what nervous systems do (i.e., descriptive models), determining how they function (i.e., mechanistic models), and understanding why they operate in particular ways (i.e., interpretive models) at multiple levels of abstraction (Dayan and Abbott, 2001). Thus, their goal is fundamentally different from data-driven approaches as they “force” us to seek mechanisms and causality (Figure 1A).

First, biophysical models of synaptic, cellular, and neural circuits aim to describe the association between psychiatric symptoms and abnormal information processing intrinsic to assemblies of neurons and microcircuit dysfunctions. These models aim to investigate mechanisms underlying cognitive decline and dementia. For instance, early work like the “synaptic deletion and compensation” model (Horn et al., 1993; Ruppin and Reggi, 1995) demonstrated that synaptic connections in Alzheimer's disease are associated with memory loss and learning difficulties. Second, large-scale neural network models address the links between psychiatric problems and information processing dysfunction intrinsic to large circuit functions (e.g., Raj and Itturia-Medina, 2019). Third, normative models address how the nervous system should behave and how certain behavior or neural activity deviates from those standards (e.g., “Perception and Attention Deficit model”; Collerton et al., 2005; Makin et al., 2013).

A concern with many theory-driven models is that they are often based upon mechanisms that are not directly accessible from experimental data (Moran et al., 2011) and provide very specific assumptions that do not lead to empirically testable predictions (Baker et al., 2018). Hence, methods that can bridge the gap between modeling the clinically relevant symptoms (at macro-level) and modeling the brain where the neurobiological mechanisms are implemented (at micro-level) are urgently needed. Here, we argue that intermedium level models would complement macro- and micro-level models, being specifically targeted at “meso-level” modeling. This allows for directly represented distributed control of neural mechanisms and neurobiologically detailed cellular functions.

At this meso-level, the model has sufficient complexity to prescribe the hierarchical architecture of the brain (i.e., a layer of units rather a single unit representing a brain region); while each layer still can implement macro-level distributed representations for perception, action, and language. Each unit in the model also includes micro-level biological details (e.g., membrane potentials, ion channels, neurotransmitters) allowing empirical validation such as by using neuroimaging. This has the potential to extend traditional models that are predominantly informative on only one level of abstraction (Figure 1B).

Examples of such models include our work on modeling attentional impairments in Alzheimer's disease and making predictions about possible electrophysiological features in the relevant neural circuits (Mavritsaki et al., 2019). These models can be seen as “virtual” patients capturing the cognitive dysfunctions on computer simulations. By testing the models with neuroimaging, biological and clinical data from real patients, we can obtain mechanistic understanding and develop new drug treatments *in-silico* before they are experimented on animals and humans. This can further speed up translational effects and drug development in an area of great unmet need, reduce socio-economic impact, and add to sustainability efforts in dementia research.

### Combining Data-Driven and Theory-Driven Computational Approaches

Combining theory-driven and data-driven approaches in a single modeling framework has the potential to account for the complexity of different forms of dementia while considering overlapping pathologies and clinical symptoms (Figure 1C). This is particularly crucial for: (I.) providing the understanding of the underlying mechanisms and “causal” interactions obtained by theoretical models, and for (II.) overcoming the current scalability limitations of theoretical models (Baker et al., 2018). For example, Maia and Frank (2011) used reinforcement learning to quantify the learning ability of individuals with Parkinson's disease based on the underlying dopaminergic mechanisms. Pinaya et al. (2021) used deep learning on normative models of brain structure to detect Alzheimer's disease progression. Bayesian models such as Dynamic Causal Modeling (DCM; Friston et al., 2016) can be applied to neuroimaging data to describe effective connectivity within and among neural circuits providing a principled data-driven way to fit subsets of model parameters in theory-driven models.

Theory-driven models provide prior knowledge and context for estimating features specifically relevant to disorders. This enables data-driven models to derive parameters for further modeling (e.g., biophysically realistic recurrent neural network models, algorithmic reinforcement learning models, Bayesian models) with increased efficiency and reliability (Huys et al., 2016). Hence, combining these approaches and linking between the levels of abstraction have the potential to increase translational benefits by relating symptoms and cognitive functions to clinically traceable entities such as cellular processes.

For instance, AI models based on recurrent neural networks are approaching human-level performance in many domains. Thus, if implemented with plausible biological details, recurrent neural networks can be “meso-level” models trained to simulate patients' clinical symptoms while the model parameters are simultaneously fitted to patients' neuroimaging and biological data. These models often contain tens of thousands of artificial neurons, making them large enough to model complex symptomology while remaining tractable to study neural mechanisms in unprecedented detail. Previous work summarized the application of such models to complex psychiatric disorders where sufficient information about the relevant circuits exist, such as in schizophrenia (Huys et al., 2016). We argue that there is potential to extend these applications to study underlying mechanisms of different forms of dementia. Additionally, by integrating computational models with neuroimaging, neuronal dysfunction underlying psychosis symptoms in dementia such as hallucinations, delusions, paranoia could be explained by impairments in multiple neurotransmitter systems (Marreiros et al., 2013). This could link clinical symptoms with biological details more comprehensively than was previously available.

## Discussion

Computational psychiatry and neurology endeavor a biopsychological and mechanistic perspective by showing how each level of abstraction ranging from molecular to circuit levels can provide a context for the human brain's hierarchical architecture, functioning and disorders. Computational approaches provide a whole new lexicon for understanding neural processes (Montague et al., 2012). Machine learning techniques can detect complex and subtle mental and brain dysfunctions and their neurobiological underpinnings that are otherwise difficult to uncover.

Computational approaches are a valuable tool moving forward in research, but the current implementation introduces several challenges. First, the availability of good quality data is crucial to create reliable, accurate, and robust data-driven and theory-driven models of mental health illnesses and brain disorders. This includes the need for widely generalisable, open access and reproducible data of different dementia types with large sample sizes (Pellegrini et al., 2018). Second, the differences in the standardization in the dementia care pathway across clinical practices, assessment centers and research might pose a further challenge in appropriate data digitalisation, curation, and integration (Wong-Lin et al., 2020). Third, researchers should additionally ensure that data pre-processing steps do not introduce unrealistic attributes to general healthcare datasets when used in modeling less-common types of dementia.

When interpreting models, researchers need to be conscious of potential challenges. For instance, predictive models in psychiatry still suffer from overfitting and lack of generalisability and validation (Meehan et al., 2022). Finding a model with the appropriate complexity, therefore, requires finding a suitable balance between bias and variance (Lever et al., 2016). Yet, testing and falsifying current models and subsequent ability to develop accurate predictions of both common and rare types of dementia are still challenging due to the limited knowledge of their neurobiology and neuropathology.

Traditional AI modeling techniques and biophysical models often consider the macro-level constraints on the brain and mind for very specific cognitive phenomena. However, existing neural models do not satisfactorily provide an architectural-level explanation for how symptoms experienced by patients were mechanistically produced by genetic, molecular, and circuit abnormalities in different inter-connected brain regions. Moving forward, we need models that do not only describe data but can also manipulate data while preserving the integrity of the data. In other words, we argue to “treat the models as if they are data”.

If present limitations are considered thoroughly, computational psychiatry and neurology models for dementia have the potential to establish as experimental medicine platform for personalized medicine and development of novel treatments and to advance the predictive health systems that would support clinicians in their decision-making process (Miotto et al., 2017). Importantly, current challenges are likely to be minimized by fast-evolving computational fields such as AI models based on deep-learning, which can serve as the basis of “virtual” patients. This allows for testing mechanistic hypotheses, while maintaining the informative value obtained from real life data.

## Author Contributions

LK and LS wrote the manuscript. SD and LJ contributed to the writing and provided feedback for the manuscript. LS secured the funding and oversaw the study. All authors have made a substantial contribution to this work and approved it for publication.

## Funding

LK was the recipient of the University of Sheffield Flagship Scholarship. LS's participation was funded by Alzheimer's Research UK Senior Research Fellowship (ARUK-SRF2017B-1).

## Conflict of Interest

The authors declare that the research was conducted in the absence of any commercial or financial relationships that could be construed as a potential conflict of interest.

## Publisher's Note

All claims expressed in this article are solely those of the authors and do not necessarily represent those of their affiliated organizations, or those of the publisher, the editors and the reviewers. Any product that may be evaluated in this article, or claim that may be made by its manufacturer, is not guaranteed or endorsed by the publisher.
